# Mechanical properties, water sorption characteristics, and compound release of grape seed extract-incorporated resins

**DOI:** 10.1590/1678-7757-2016-0448

**Published:** 2017

**Authors:** Don Jeevanie EPASINGHE, Cynthia Kar Yung YIU, Michael Francis BURROW

**Affiliations:** 1 Prince Philip Dental Hospital, University of Hong Kong, Faculty of Dentistry, Hong Kong SAR, China.; 2 University of Melbourne, Melbourne Dental School, Melbourne, Australia.

**Keywords:** Grape seed extract, Elasticity, Solubility, Resins, Hardness

## Abstract

**Objective:**

This study evaluated the effect of grape seed extract (GSE) incorporation on the mechanical properties, water sorption, solubility, and GSE release from the experimental adhesive resins.

**Material and Methods:**

An experimental comonomer mixture, consisting of 40% Bis-GMA, 30% Bis MP, 28% HEMA, 0.26% camphorquinone and 1% EDMAB, was used to prepare four GSE-incorporated adhesive resins at concentrations of 0.5, 1, 1.5, and 2 wt%. The neat resin without GSE was used as the control. Six resin beams (25 mm x 2 mm x 2 mm) *per* group were prepared for flexural strength and modulus of elasticity evaluations using a universal testing machine at a crosshead speed of 1 mm/min. Five disks (6 mm in diameter and 2 mm in thickness) per group were used for microhardness measurements using a Leitz micro-hardness tester with Leica Qgo software. Five disks (7 mm in diameter and 2 mm in thickness) per group were prepared and stored in deionized water for 28 days. Water sorption, solubility, and GSE release in deionized water were calculated for each GSE-incorporated adhesive at the end of 28th day. Data was evaluated using one-way ANOVA and Tukey multiple comparisons.

**Results:**

Flexural strength, modulus of elasticity and microhardness of GSE-incorporated adhesive decreased significantly with incorporation of 1.5% of GSE (p<0.05). Addition of GSE had no effect on the water sorption of the adhesive resins (p=0.33). The solubility of the resin also increased significantly with incorporation of 1.5% of GSE (p<0.05). Quantities of GSE release increased with increased concentration of GSE in the adhesive resin.

**Conclusion:**

Up to 1% of GSE can be incorporated into a dental adhesive resin without interfering with the mechanical properties or solubility of the resins.

## Introduction

In the modern world, the use of tooth-coloured resin restorations has become very popular, resulting in an increased use of resin adhesives. However, these aesthetic bonded restorations are less durable than the traditional amalgam fillings^8^. The resin-dentine bonded interface is subjected to various mechanical forces, chemical or enzymatic challenges and deteriorates over time^24^. The ability of the bonded interfaces to resist these challenges will increase the longevity of the aesthetic bonded restorations^27^.

Several attempts have been made to increase the durability of bonded restorations either by modifying the resin or the dentine substrate^30^. Complete infiltration of the demineralized collagen fibrils by adhesive resins is difficult to achieve^19^. The unprotected collagen fibrils in the hybrid layer are exposed to enzymatic challenges by proteases either from exogenous or endogenous origins^18^. Various matrix metalloproteinase (MMP) inhibitors and collagen cross-linkers have been introduced as pre-conditioning agents to preserve the stability of the collagen and enhance long-term durability of the bonded interface^7^.

Recently, grape seed extract (GSE) has shown beneficial effects in the preservation of collagen fibrils. Grape seed extract contains mostly proanthocyanidins. Thus, GSE acts as a collagen cross-linker, MMP inhibitor, and remineralizing agent^3,4,22,28^. The use of GSE or proanthocyanidin as a pre-conditioning agent on dentine has improved the resin-dentine bond strength of both sound and caries-affected dentine^1^. The effects of proanthocyanidin on the mechanical properties of dentine remain high up to three months, but decrease after six months^20^. Proanthocyanidin from GSE has been used as a pre-conditioning agent in various concentrations for durations of 10 minutes to 1 hour^1,3^. The prolonged application time is clinically impractical; hence, proanthocyanidin in the form of GSE has been incorporated in an adhesive resin to simplify the bonding procedures and to allow for a sustained release of GSE in the demineralized dentine over time^6^.

Several studies have incorporated GSE or proanthocyanidin in the adhesive resins and have shown beneficial outcomes^6,9^. The incorporation of 2.5% of GSE in an adhesive preserved the collagen matrix against collagenase stress for 6 days; by contrast, the collagen fibrils in the bonded interfaces formed by an adhesive without GSE were degraded^9^. Studies have shown that up to 2% of proanthocyanidin from GSE can be incorporated into experimental etch-and-rinse adhesives without adversely affecting bond strength^6^. Therefore, before examining the effect of a GSE-containing adhesive on the durability of resin-dentine bonds, it is necessary to examine whether the incorporation of GSE into the adhesive resin and its subsequent release affects its mechanical properties. Hence, this study examined the effects of incorporation of low concentrations of GSE on the mechanical properties, water sorption, solubility, and GSE release of an experimental adhesive resin. The null hypotheses tested were that GSE incorporation has no effect on (i) the mechanical properties of the adhesive resins; (ii) the water sorption and solubility of the adhesive resins, and (iii) the rate of GSE release from the cured resins.

## Material and methods

### Experimental resins preparation

A blend of methacrylate resin comonomers consisting of 40 wt% bisphenol A diglycidyl ether dimethacrylate (Bis-GMA), 30 wt% Bis[2-(methacryloyloxy)ethyl] phosphate (Bis-MP), 28.80 wt% 2-hydroxylethyl methacrylate (HEMA), 0.26 wt% camphorquinone, and 1 wt% ethyl N, N-dimenthyl-4-aminobenzoate (EDMAB) was used to formulate the four experimental GSE-incorporated adhesive resins. Grape seed extract (>95%, Oligomeric Proanthocyanidin, International Laboratory of USA, South San Francisco, CA, USA) was added to the neat comonomer blends at 0.5, 1, 1.5, and 2 wt%. The neat comonomer blend without GSE was used as control group.

### Specimen preparation for three-point bending test

The experimental resins from each test group were placed in 25 mm x 2 mm x 2 mm brass molds without allowing any air entrapment. The molds were sandwiched between two glass slabs covered with polyethylene films to eliminate oxygen inhibition layers on the cured resin surfaces. The resins were light-cured on both sides of the specimen with a quartz-halogen light curing unit (Elipar TM 2500, 3M ESPE, St. Paul, MN, USA) operated at 600 mW/cm^2^ for 3x40 s. After curing, the resin specimens were removed carefully from the mold and checked under a stereomicroscope for the presence of air bubbles or cracks. Specimens containing voids or cracks were discarded. The specimens were then polished with 360-grit silicon carbide paper to smooth the rough edges. Six specimens were prepared from each group of dental adhesive resin containing 0, 0.5, 1, 1.5, and 2 wt% of GSE. The specimens were stored in dry conditions at 37°C for 24 h before testing.

### Three-point bending test

Three-point bending test was performed using a Universal testing machine (ElectroPlus^TM^ E 3000, Instron Industrial Products, Grove City, PA, USA) at a crosshead speed of 1.0 mm/min. A span length of 20 mm was used. Before testing, the dimensions of the specimens were determined using a digital caliper (Mitutoyo Corp, Tokyo, Japan) to the nearest 0.01 mm. Load deflection curves were recorded and plotted to obtain the slope for linear portion of the curve. The maximum load and the slope of the curve were used to evaluate the flexural strength and the modulus of elasticity.

Flexural strength (FS) and Modulus of elasticity (MoE) were calculated using the following formulas^13^:

Flexural strength=3FL/2bh^2^

Modulus of elasticity=SL^3^/4bh^3^

F: maximum load in load deflection curve (N)

L: span between two supports (mm)

b: width of the specimen (mm)

h: height of the specimen (mm)

S: Slope of the linear portion of the load deflection curve

### Vickers hardness test

Five samples of resin disks were prepared from each resin group using a brass mold of 6 mm in diameter and 2 mm in thickness. The top and the bottom of the brass mold were covered with glass slides and kept in a non-reflective flat surface during curing. The cured specimens were removed from the mold and aged for 24 h at 37°C dry and dark chamber. Leitz micro-hardness tester (Leica, Tukon 200, Germany) with Leica Qgo software was used to perform the microhardness evaluation of the resin disks. All readings were obtained from the surface of the specimen. A load of 0.5 N was applied for 20 s. A total of 10 readings were obtained from each group of adhesive resin.

### Water sorption and solubility

Five disks (12 mm ± 0.1 mm in diameter and 0.7 mm ± 0.1 mm in thickness) were prepared for each tested adhesive resin using a Teflon split ring mold. To avoid the oxygen inhibition layers, two glass slides covered with polyethylene films were used to seal the two sides of the mold. The resin was carefully placed into the mold avoiding any bubble formation and light-cured at the center of the disk for 40 s and at its opposite side for 40 s using a quartz-tungsten-halogen light-curing unit operated at 600 mW/cm^2^.

Each polymerized resin disk was kept in a silica gel-containing desiccator and weighed on an analytical balance (Model AD6, Perkin Elmer, Shelton, CT, USA) until a constant mass was obtained (M1). Each disc was individually stored in 5 mL of deionized water at 37°C in a sealed container for 4 weeks. The specimen was taken out from the solution after 3, 12, 24, 48, 72, 96, 120, 144, 168, 240, 288, 336, 508, and 678 h (28 days), blot dried, mass change recorded, and re-immersed in the respective solution after measurement. The maximum mass (M2) was obtained during the 28-day experiment. After completing 28 days of water storage, each specimen was placed in a desiccator and weighted daily until a constant mass (M3) was reached. The percentages of water sorption (WS) and solubility (S) were calculated using the following formulas^15^:

WS=M2-M1/M1

S=M1-M3/M1

### Grape seed extract release

Reference solutions containing 0.1, 0.2, 0.5, 1, 2, 5, 10, 20, 50, and 100 ppm of GSE solutions dissolved in deionized water were prepared to achieve a linear pattern between the amount of GSE and absorbance peak height. Maximum absorbance peak for GSE was confirmed as 278 nm in the UV-Vis spectrophotometer, which was stated in previous studies. Fifteen GSE-incorporated resin disks were prepared as previously described and stored in deionized water. The diameter and the thickness of each specimen were measured using digital calipers (Mitutoyo Corp, Tokyo, Japan) to the nearest 0.01 mm. At appropriate time points, the UV absorbance (LAMBDA 950 UV/Vis/NIR Spectrophotometer, PerkinElmer, USA) of the deionized water was measured and converted to the quantities of GSE released using the linear relationship obtained. The rate of GSE release (mg) *per* unit area of the resin disk (µm^2^) was calculated between consecutive measurements and the cumulative release (mg) *per* gram of the sample was obtained for the 28-day period^26^.

### Statistical analyses

Data were analysed using a statistical package (SigmaStat Version 16, SPSS, Chicago,USA). The normality (Kolmogorov-Smirnoff test) and homoscedasticity assumptions (Levene test) of the FS, MoE, microhardness, and cumulative GSE release data appeared to be valid. One-way ANOVA was used to examine the effect of GSE concentration on FS, MoE, and microhardness. Two-way ANOVA was used to evaluate the effect of “GSE concentration” and “storage time” on the GSE release rate. *Post-hoc* multiple comparisons were carried out using the Tukey test, with significance set at p<0.05.

## Results

### Modulus of elasticity, flexural strength, and microhardness

Table 1 shows the changes of MoE, FS, and microhardness of the experimental resins with different concentrations of GSE. One-way ANOVA and Tukey multiple comparisons showed that “GSE concentration” significantly affected the FS (p=0.000), MoE (p=0.000), and microhardness (p=0.000) of the adhesive resins (p<0.001). The addition of 1.5 and 2% GSE significantly reduced the FS, MoE, and microhardness of the resins (p<0.001). The addition of lower concentrations of GSE (0.5-1%) had no effect on the FS (p=0.73, p=0.75), MoE (p=0.99, p=0.87), and microhardness (p=0.17, p=0.67) of the experimental resins (p>0.05).

**Table 1:** Flexural strength, modulus of elasticity, and Vickers Hardness of grape seed extract-incorporated resins

GSE Conc	FS (MPa)	MoE (GPa)	Vickers Hardness
0%	19.11±1.73^a^	0.5±0.18^a^	10.29±1.59^a^
0.5%	17.57±3.40^a^	0.42±0.24^a^	9.13±1.13^a^
1%	17.52±2.94^a^	0.48±0.13^a^	8.89±1.09^a^
1.5%	8.15±0.93^b^	0.12±0.02^b^	4.23±0.48^b^
2%	6.30±0.98^b^	0.08±0.02^b^	5.43±1.19^b^

FS: Flexural Strength; MoE: Modulus of Elasticity; VH: Vickers Hardness

Values are expressed as means and standard deviations.

Groups identified by different superscript letters are statistically significant (p<0.05)

### Water sorption and solubility

Table 2 shows the mean and standard deviation of the water sorption and solubility of GSE-incorporated resins. One way ANOVA and Tukey multiple comparisons showed that GSE concentration had no effect on the water sorption of GSE-incorporated resins (p=0.33), but significantly affected (p=0.000) the solubility of the GSE-incorporated resins (p<0.001).

**Table 2:** Water sorption and solubility of grape seed extract-incorporated resins

GSE Conc	WS%	S%
0%	9.04±1.96^a^	5.24±1.49^a^
0.5%	7.89±1.95^a^	5.02±0.97^a^
1%	8.48±1.45^a^	4.58±1.04^a^
1.5%	7.64±2.48^a^	9.96±2.53^b^
2%	8.72±2.33^a^	10.72±3.45^b^

WS: water sorption; S: solubility

Values are expressed as means and standard deviation.

Groups identified by different superscript letters are statistically significant (p<0.05)

### Proanthocyanidin release

**Figure 1:**
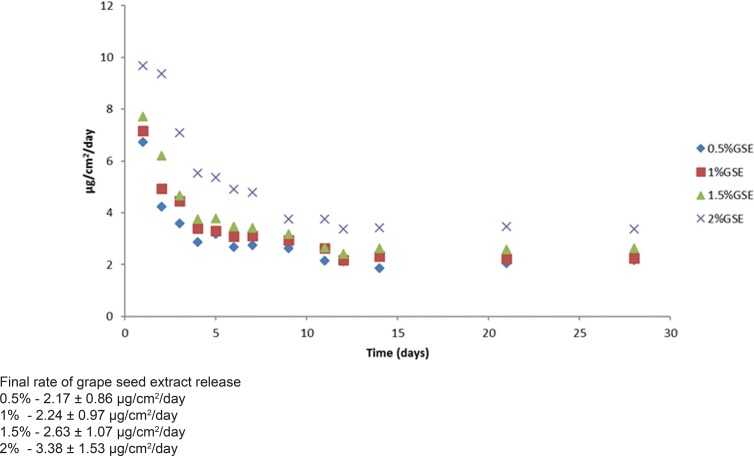
- Rate of grape seed extract release from grape seed extract-incorporated adhesive resins over 28 days

**Figure 2:**
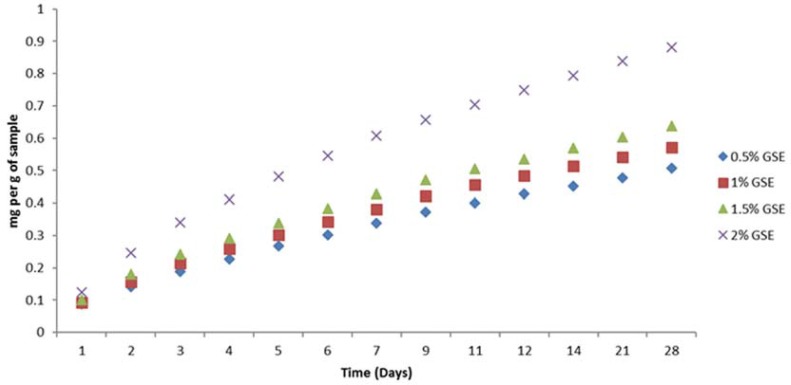
Cumulative release of grape seed extract from grape seed extract-incorporated adhesive resins within 28 days

The rates of GSE release from the GSE-incorporated adhesive resins are shown in Figure 1. No release of GSE was observed from the control resin. Two-way ANOVA showed that both “GSE concentration” and “storage time” affected (p=0.000) the GSE release rate (p<0.05). In general, after an initial burst of GSE release for 48 h, the mean release rate declined rapidly and reached a stable plateau after 5 days. The order of GSE release rate is 2%>1.5%>1%>0.5%. The cumulative GSE release increased with the concentration of GSE in the adhesive resin and was the highest in the 2% GSE-incorporated adhesive (Figure 2).

## Discussion

The addition of a therapeutic material into dental adhesive resin can disturb its polymerization and affect the mechanical properties of the polymerized resin^2^. Grape seed extract is a source of proanthocyanidins and, during our study, we have used GSE containing 90-95% of proanthocyanidins. Since our aim was to use the proanthocyanidin from GSE, we have used the GSE available with the highest concentration of proanthocyanidin for the laboratory studies. As GSE is a free radical scavenger, it is necessary to find an optimum GSE concentration for incorporation into dental resins, so as to maximize its cross-linking and protease inhibitory properties, with minimal adverse effects on resin polymerization properties. However, it was difficult to select the appropriate GSE concentrations, since proanthocyanidin from GSE has been used in previous studies at a higher concentration as a pre-treatment agent^3^. Recently, it has been shown that the incorporation of 2.5-10% of GSE reduced the degree of conversion of the adhesive resins^16^. Thus, we have incorporated a lower concentration of GSE (0-2%) into adhesive resin and evaluated the effects of GSE incorporation on the mechanical properties, water sorption, and solubility of the adhesive resins, as well as GSE release from the resin.

The flexural strength, modulus of elasticity, and microhardness were all adversely affected by the incorporation of 1.5 and 2% GSE. The first null hypothesis that GSE concentration had no effect on the mechanical properties of adhesive resin was rejected. GSE contains oligomeric proanthocyanidin, which is a plant flavonoid, belonging to the flavanol group. It is a known anti-oxidant, which acts as a terminator of free radical reactions^12^. Thus, GSE can inhibit the polymerization of the resin by deprotonating the OH groups in their molecules^12^.

Camphorquinone (CQ) and amine system were used in this study as the photosensitizer and hydrogen-donating co-initiator of the experimental adhesive resins^10^. With light curing, CQ absorbs the light energy and is converted to an excited unstable status, which extracts hydrogen atoms from the tertiary amine. This results in the formation of aminyl free radical, which initiates the chain polymerization. The proanthocyanidin from GSE, when involved in the radical polymerization, donates hydrogen atoms to the free radicals and inhibits the initiation and propagation of the chain reaction^16^. Even with the incorporation of lower concentrations of GSE into the adhesive resins, some disturbances to free radical polymerization might have occurred, as shown by the slight reduction in flexural strength and modulus of elasticity with the addition of 0.5% - 1% GSE. However, with the incorporation of 1.5 and 2% GSE, the GSE concentration reached a threshold and the chain polymerization was inhibited, consequently jeopardizing the mechanical properties of the adhesive resin.

The effect of GSE concentration on microhardness also followed a similar pattern. In general, a reduction of Vickers Hardness was observed with the addition of GSE into the adhesive resin. This reduction became significant when the GSE concentration was 1.5% and higher. Microhardness values were considered to be more sensitive to small changes in the polymer chain formation and propagation^5,21^. Due to the interferences of GSE on free radical polymerization, the hardness of the GSE-polymerized resin was reduced. Grape seed extract contains the oligomeric proanthocyanidin molecule and the higher molecular size can further interrupt chain propagation by separating the monomer molecules from the continuing polymer chain, thereby reducing the integrity of polymerized material^2^.

Moreover, the altered light intensity of the resin with the incorporation of higher concentrations of GSE may be another mechanism that affects the resin polymerization. The increased colour intensity with GSE incorporation might affect the penetration of light and reduce the depth of cure incrementally. It has been shown that different shades of composite have an effect on the depth of cure and eventually on the degree of polymerization of the resin^11^. Furthermore, GSE is mildly acidic, and the acidity of the adhesive resin may be increased with its addition. The formation of an insoluble salt with the co-initiator may occur as a result of increasing acidity of the resin mixture^10^. Hence, only a low concentration of GSE should be incorporated into the adhesive resin to avoid these detrimental effects on mechanical properties.

The incorporation of hydrophilic materials, such as GSE, can cause water-filled droplets around GSE molecules within the polymer resin matrix^25^. These droplets increased in size along the osmotic gradient between the droplets and the external solution^25^. Equilibrium is obtained when the polymer elastic and osmotic stresses balance each other. However, with the increased hydrophilicity of the adhesive resin, this equilibrium can be disturbed. The increased water sorption by the hydrophilic resin affected its mechanical properties, resulting in a weaker resin^29^. Thus, it is important to evaluate the water sorption of the adhesive resin with and without GSE.

There was no significant difference in water sorption between the different GSE-incorporated adhesive resins. However, the solubility of the GSE-incorporated adhesive resins was adversely affected by the addition of 1.5 and 2% of GSE. Hence, the second null hypothesis that GSE incorporation had no effect on the water sorption and solubility of the adhesive resins was partially rejected. Only a low concentration (0-2%) of GSE was added to the adhesive resin, thus the water sorption of the GSE-incorporated adhesive resins is mainly attributed to the hydrophilicity of the resin itself rather than the GSE incorporated. According to Sideridou, et al.^23^ (2003), solubility depends on the amount of unreacted monomers trapped within the polymer matrix. The presence of unreacted monomers depends on the degree of conversion of the resin. Hence, a higher solubility is an indirect indication of the lower degree of conversion of the adhesive resin. The significant increase in solubility with the incorporation of 1.5% of GSE into adhesive resin can be interpreted as a sign of lower degree of polymerization of the resin.

This study aimed to examine the possibility of incorporation of GSE into an adhesive resin to enhance the biomechanical properties of the dentine by providing sustained release of GSE over time. Therefore, the quantity of GSE release from the GSE-incorporated adhesive resins over 28 days was examined. Our results showed that incorporation of 2% of GSE into the resins has the highest amount of GSE release when compared to 0.5-1.5%. Thus, the third null hypothesis that GSE incorporation had no effect on the rate of GSE release from the cured resins was rejected. All the tested adhesives showed an initial burst of GSE release in the first 24 h, and this was previously reported to be due to the presence of microvoids and surface-bound drugs^14^. However, the GSE release became stable after 5 days and remained static for the rest of the observation period. Being an oligomeric molecule, GSE release from the polymer matrix may be difficult. Nonetheless, the hydrophilicity of the resin allows bulk water movement into the resin, facilitating the release of water-soluble GSE^17^. Further studies are necessary to evaluate the effect of GSE incorporation into adhesive resin on the durability of the resin-dentine bond.

## Conclusions

Within the limits of this study, it may be concluded that:

The incorporation of 0.5 to 1% grape seed extract into an adhesive resin had no adverse effect on its mechanical properties, water sorption, and solubility.

The rate of grape seed extract release increased with the concentration of grape seed extract incorporated into the adhesive resin.
